# A Medical Resident with a History of Alcohol Abuse and Suicidal Ideation: A Challenge for Both Psychiatry and Occupational Medicine in the Context of the First Wave of the COVID-19 Pandemic

**DOI:** 10.1155/2022/7396453

**Published:** 2022-10-17

**Authors:** Martina Corsi, Antonello Veltri, Salvio Perretta, Riccardo Marino, Gabriele Necciari, Fabrizio Caldi, Rudy Foddis, Alfonso Cristaudo, Rodolfo Buselli, Giovanni Guglielmi

**Affiliations:** Occupational Health Department, Azienda Ospedaliero-Universitaria Pisana, 56124 Pisa, Italy

## Abstract

This case study draws attention to the hazards of physicians with a history of alcohol addiction and a particular psychopathology framework in the context of occupational health surveillance, particularly during the challenging working conditions brought about by the COVID-19 pandemic. The case involves a hospital resident in her thirties, with a previous history of addiction and attempts at suicide, who was assigned to a COVID-19 unit of an Italian hospital. In this case study, we discuss the preventive intervention put in place in order to protect physicians' health and work. What emerges is the key role that rapid substantive communications between specialists play in formulating an effective strategy for dealing with these conditions. We believe this case is noteworthy for the lessons that can be learned for tailoring prevention and treatment pathways for health care workers with addiction.

## 1. Introduction

Alcohol is one of the few psychotropic drugs, with intoxicating and dependence-producing properties, that has had its use acknowledged and encouraged by society [1]. Accumulated evidence indicates that alcohol consumption is associated with inherent health risks, although health consequences of alcohol consumption vary significantly in magnitude and nature among drinkers [2–4]. According to the World Health Organization (WHO), the mortality rate of alcohol consumption is 4 per cent and the rate of resulting functional limitations is between 4 and 5 per cent [2]. Alcohol use is the third largest risk factor for around 60 diseases and disabilities, including different cancers, cirrhosis of the liver, cardiovascular diseases, and epilepsy. Heavy drinkers are also at greater risk of conditions such as hypertension and gastrointestinal bleeding. Alcohol use is also responsible for the exacerbation of certain mental disorders including sleep disorders and depression. Additionally, alcohol use is also associated with poor social outcomes such as relationship breakdown, trauma, violence, child neglect and abuse, and workplace absenteeism [5].

A worker's decision to drink is of course a personal decision. However, when the use or abuse of alcohol interferes with the worker's ability to perform his or her duties, this can become a public health concern [6].

It may often be assumed that health professionals have a healthier lifestyle than the rest of the population, but this is not necessarily true [7]. On the contrary, health care professionals may be more susceptible to alcohol abuse because of their typically stressful jobs, burnout, heavy workloads, and frequent contact with illness and death [8]. A large body of evidence suggests that a significant proportion of health professionals has high rates of alcohol use [9], with consumption increasing over time [6, 10]. In a review paper, Baldisseri estimated that around 14% of doctors have an Alcohol Use Disorder (AUD) [11]. The two main international clinical diagnostic systems, the ICD published by the World Health Organization (WHO) and the Diagnostic and Statistical Manual of Mental Disorders, Fifth Edition (DSM-5) published by the American Psychiatric Association, define a series of disorders related to alcohol consumption. These are of two conceptual types: those that denote a pattern of repetitive alcohol consumption and those that reflect the harmful consequences of that consumption [12]. DSM-5 defines AUD as a medical condition characterized by an impaired ability to stop or control alcohol use despite adverse social, occupational, or health consequences [12, 13]. The ICD-11 presents a broader approach to alcohol consumption ranging from hazardous pattern of alcohol use to harmful pattern of alcohol dependence [12].

Among physicians, anesthesiologists have more problems with psychoactive substance abuse, making it the main occupational hazard in this group [14, 15]. Nevertheless, studies evaluating the use of alcohol by health care professionals are scarce, and there are often underestimated data on this matter owing to the tendency to disregard the problem, caused by the fear of consequences in the work sphere and professional status [6, 8, 16, 17]. Alcohol use and abuse among doctors is a delicate topic but one that is of utmost importance because it may seriously influence and even compromise the practice conducted by medical professionals [18].

In this regard, the aim of this case report is to draw attention on this topic. Italian legislation identifies the occupational physician as a key figure to prevent alcohol abuse in some work activities, but lots of difficulties in its application remain [19, 20]. We believe that highlighting real clinical experience could help other local institutions in a perspective of evidence-based practice to face this particular occupational hazard [21–23]. The final goal is to finalize tailored prevention and treatment pathways for health care workers with addiction.

## 2. Case Presentation

XX is a medical resident aged between 30 and 40 years old. She presented a cyclothymic temperament with traits belonging to borderline personality disorder. When she was a child, she experienced a traumatic event of loss as she witnessed the sudden death of a parent. XX then developed feelings of emptiness; fear of abandonment from other attachment figures; emotional swings with quick change to sadness, anger, and anxiety; or impulsive behaviours that led to a first suicide attempt by drug ingestion at the age of 14.

In the following years, the subject continued to experience mood swings. These were mainly depressive in nature with a subsequent compromission of her interpersonal skills though not of her intellectual performance. The patient maintained a very strong academic performance in school. In her last year of medicine, around the age of 25, under pressure for exams and the required final thesis, the subject developed a depressive episode according to DSM-5, characterized by low mood, loss of enjoyment in most activities, anxiety, hypoergy, difficulty with concentration, and insomnia which led to a great increase in alcohol use together with benzodiazepines. She turned to a psychiatrist and started various antidepressants (including citalopram, venlafaxine, and duloxetine). However, the clinical benefits of these antidepressants were poor, and she continued to have mood swings alternating between moments of sadness and pervasive anxiety and moments of increased work performance, self-confidence, increased involvement in hobbies as writing poetry and reading, more energy, and decreased need for sleep. Alcohol abuse remained stable through the alternation between the various mental states.

Two years later, during her first year of residency program, she presented a worsening of the psychopathology framework developing a mood episode with mixed features according to DSM-5. During that period, she started a binge use of anesthetics (midazolam, propofol), and she reached a total deterioration of general personal functioning. This was followed by hospitalization for about two weeks and discharge with a diagnosis of Bipolar Disorder (BD) and Alcohol Use Disorder (AUD) and a pharmacological treatment with valproic acid (immediately suspended by the patient herself), anxiolytic stabilizers (gabapentin), atypical antipsychotics (quetiapine), and benzodiazepines (lorazepam), again however with poor clinical benefit. During the next two years, she continued to experience mixed-depressive symptoms, despite the therapeutic attempts, which led to several hospitalizations and, at the age of 28, to another suicide attempt by means of lithium salt ingestion with intoxication that led to temporary cardiac arrest. In the following years, she continued to show mood instability that she managed by herself, combined with a daily use of alcohol that allowed her to recover her ability to work and thus return to her residency program. Nevertheless, she presented a poor adaptation on the social and leisure plan with no social circle and a lack of confidence in social situations.

In 2018, following a new worsening of the psychopathological elements with a daily alcoholic consumption of about two to three beers a day, she attempted suicide again by means of a massive ingestion of her psychiatric treatment. She was again hospitalized and started a new therapeutic program based on buprenorphine (now at 8 mg/day), various mood stabilizers then discontinued, benzodiazepines: clonazepam (later discontinued) and currently delorazepam (now 4 mg/day), tricyclic antidepressants (amitriptyline 75 mg/day), and antidepressant SARI (trazodone, 75 mg/day). Since then, the patient has shown a slight improvement in her mood and was admitted for some weeks to a therapeutic community for alcoholics from which she reported that she emerged detoxified. In 2019, she continued her residency with relative stability and good performance. In March 2020, with the outbreak of the COVID-19 pandemic, she was assigned to a COVID-19 emergency unit.

**Figure 1:**
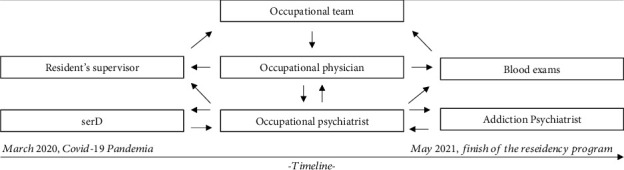
Intervention scheme.

At that point, due to various risk factors and vulnerabilities for an alcohol abuse and psychiatric relapse, the patient asked for support to deal with new stressful situations present in the workplace. As a result, the subject's case was assigned to the multidisciplinary group of the Occupational Medicine Unit (see Figure 1).

Blood count tests showed no signs of recent alcohol abuse though the subject's liver was slightly compromised. The subject denied any kind of alcohol and recent drug use except for smoking. She reported feelings of anxiety and frustration because of exclusion from procedures of her level of expertise due to her psychiatric history. Evaluation revealed that the subject suffered from a very low mood with general flattening, detachment, and difficulty concentrating. The patient did however deny suicidal ideation. At the time of the evaluation, she presented a working function within acceptable limits but still had difficulty in organizing daily life and suffered from poor social relations. She had no friends at work or outside and no emotional relationships. Our group confirmed the diagnosis of BD (according to DSM-5) with previous AUD and some features of borderline personality.

XX was offered a personal plan of monitoring of alcoholemia and periodic blood tests together with psychiatric evaluations and the possibility of new pharmacological treatment. The occupational psychiatrist (with a specialty both in psychiatry and psychotherapy) managed psychological and psychiatric interventions.

XX presented borderline personality traits with bipolar depression symptoms and posttraumatic stress elements due to the nature of the work environment brought about by the COVID-19 pandemic. She presented a flattened life likely due to prolonged use of alcohol and benzodiazepines. Consistent with the complex clinical elements of XX, previous addictive behaviours, suicidal ideation, drug resistance, and her professional role, the need to prescribe and manage off-label medications was evident [24–26]. In accordance with these considerations, in agreement with XX and with the occupational physician, the occupational psychiatrist requested a consultation with the service provided for persons with pathological addictions (*Servizi Dipendenze: SerD*) and an addictology psychiatrist. The final maintaining treatment was the following: buprenorphine [27, 28], lithium [29], methylphenidate [30, 31], and delorazepam [32] together with weekly sessions of third generation of Cognitive Behavioural Therapy (CBT) with integration of Mindfulness and Acceptance and Commitment Therapy [33] (see Table 1). The occupational team, with the consent of XX, informed her supervisor about the surveillance program and the psychiatric treatment choice and, with his assistance, drew up a new flexible working plan, more appropriate to XX's level of experience but with assistance and monitoring of her hazards.

During the following year, XX followed the Occupational Unit's indicated treatments and was able to maintain her stability and finish her residency program.

## 3. Discussion

The case of XX is interesting because it represents a difficult clinical issue that occupational teams (in this case an occupational physician and psychiatrist) have to face in common practice: substance and/or alcohol abuse among health care workers. The occupational multidisciplinary team had some challenges and targets. First is the treatment of the patient's clinical problems, protecting her work as a resident by preventing her from interrupting her specialty program or causing problems in the work during the COVID-19 pandemic.

Article 41 of Law Decree 81/08, amended and integrated by Law Decree 106/09, introduced the mandatory control of alcohol use and abuse for some categories of workers who carry out risky activities or can cause damage to the health of third parties [34, 35]. Although the scientific evidence for the existence of the problem exists, there remains a lack of attention to the potential magnitude and seriousness of this issue. Consistently, we believe that a realistic and effective solution should be sought not only in the application of the legislation but by improving occupational multidisciplinary teams [22, 24, 36, 37].

Nevertheless, detection and care of physicians with risky alcohol use is complex in daily practice, and to set up an efficient prevention strategy as well as support management for physicians is not easy. The three widely approved medications for AUD (i.e., disulfiram, acamprosate, and naltrexone) are both modestly effective and underutilized. The heterogeneity inherent in AUD also presents the issue that no one medication is likely to be effective for all individuals with AUD. Furthermore, physicians tend to specifically neglect their health and do not readily consult their colleagues. According to this, there is a need for the development of novel, diverse, and effective pharmacological treatment options with the hopes of increasing utilization and improving care [23].

A web-based Italian national survey of around 700 physicians evidenced that 15% reported increased alcohol consumption during the first wave of the COVID-19 pandemic [38]. A similar result was highlighted from another Italian survey of about 1800 health care professionals across Italy [39]. Other international studies indicate that specifically younger working women with a higher education tend to pick up more male patterns of alcohol use, putting them at risk for alcohol-related health consequences [40]. However, there are very few and recent representative studies of the drinking patterns of female doctors [19].

1^st^-level health assessment in the region of Tuscany (Decree 9 December 2013 n. 1065 regarding guidelines for health assessments of the absence of alcohol dependence in workers assigned to tasks involving particular risks for the safety and health of third parties) [34, 35] is characterized by occupational physician interviews aimed at identifying acute and chronic alcohol-related problems, blood sampling, or even not informed alcohol test and possible collaboration with other specialist subjects after informed consent, and that is what was offered to XX.

A further notable feature of this case study lies in the off-label psychiatric treatment choice alongside the CBT program. Off-label drug use refers to the use of drugs outside the conditions of the product license in terms of dose, patient age, route of administration, indications, and contraindications and is relatively common in medical practice, even if it is not often supported by strong scientific evidence [25, 41]. Despite the availability of many classes of psychotropic drug, significant numbers of psychiatric patients remain troubled by distressing and disabling symptoms even after a succession of licensed pharmacological treatments, so psychiatrists may then consider the prescription of a psychotropic outside the narrow terms of its license, as part of an overall management plan especially for the so-called “orphan” conditions as in this specific case where there were a significant number of borderline personality traits [42]. Consistent with this, in this case report, the treatment choice was based on the need to answer primarily preventive clinical questions: avoiding alcohol craving and depressive as well as suicidal ideation relapses which could have had a negative effect on both the health and work performance of XX. In the framework of the CBT approach, positive psychology techniques have been integrated to motivate XX to see herself as skillful and as having capabilities and resources to spend to reduce pain and suffering, to resolve concerns and conflicts, and to empower her resilience and more efficiently cope with work stress [43].

## 4. Conclusion

This clinical experience has led us to consider the importance of personalized prevention and intervention programs. The clear and rapid communication between the occupational physician, psychiatrist, and resident's supervisor and the use of shared choices allowed the team to protect the health and work of the physician.

## Figures and Tables

**Table 1:** Psychiatric treatment rationale.

Service for pathological addictions: serD	Occupational psychiatrist	Addictology psychiatrist
(i) Confirmed low dose of *buprenorphine*: a partial mu-opioid receptor agonist and an antagonist of the kappa opioid receptor used to treat opioid dependence in order to reduce cravings and drug-seeking behaviours. Further, several pieces of evidence demonstrate that it is an efficacious, well-tolerated, and safe option in reducing depressive symptoms and serious suicidal ideation in treatment-resistant depression	(i) Added *lithium*: a drug with intrinsic antisuicidal property in order to prevent suicide attempts (ii) Stopped *trazodone*: no beneficial effects on sleep pattern (iii) Confirmed *delorazepam*: it could not be removed in this phase because the patient had been doing it for a long time for anxiety and alcohol addiction: further, the patient already tried acamprosate and sodium oxibate without a benefit on craving (iv) *CBT*: to increase patient insight and to ensure continuity of the therapeutic project	(i) Stopped *amitriptyline*: no beneficial effects even at high doses. Already used many classes of antidepressants (ii) Added a low dose of *methylphenidate*: stimulants which promote alertness and wakefulness and potentially enhance cognitive function appear to be potential treatment options in bipolar-depressed subjects not responding to first-line treatment

## Data Availability

Data sharing is not applicable to this article as no new data were created or analyzed in this study as it is a case report. Some data could be available on request due to privacy/ethical restrictions
